# Effectiveness and equity of mHealth apps for preeclampsia management in LMICs: A rapid review protocol

**DOI:** 10.1371/journal.pone.0313655

**Published:** 2024-11-13

**Authors:** Abdirahman Moallim Ibrahim, Shayesteh Jahanfar

**Affiliations:** 1 SD Gupta School of Public Health, IIHMR University, Jaipur, India; 2 Faculty of Medicine & Surgery, Jazeera University, Mogadishu, Somalia; 3 Department of Public Health and Community Medicine, Tufts School of Medicine, Boston, MA, United States of America; The Hong Kong Polytechnic University, HONG KONG

## Abstract

**Introduction:**

Preeclampsia remains a formidable public health challenge, particularly in low- and middle-income countries (LMICs), where it significantly contributes to the high rates of maternal and neonatal morbidity and mortality. The advent of mobile health (mHealth) applications presents a promising avenue for enhancing the management of preeclampsia. This review protocol is designed to systematically assess the effectiveness and equity of mHealth apps in managing preeclampsia within LMICs, with a focus on clinical outcomes and the broader implications for accessibility, affordability, and cultural relevance.

**Materials and methods:**

To achieve the objectives of this review, a rapid review methodology will be employed, encompassing a structured search strategy to identify pertinent studies from databases such as PubMed, Cochrane Library, and Google Scholar, as well as grey literature. The inclusion criteria are set to encompass randomized controlled trials (RCTs), controlled clinical trials (CCTs), observational studies, and qualitative studies that offer insights into the effectiveness and user experience of mHealth apps for preeclampsia management. Participants in these studies will include pregnant women at risk for or diagnosed with preeclampsia, healthcare providers, and app developers. The quality of the included studies will be critically appraised using standardized tools, and data extraction will focus on study characteristics, interventions, outcomes, and equity considerations.

**Discussion:**

The implications of this review are far-reaching, offering the potential to inform stakeholders including policymakers, healthcare providers, and app developers about the deployment and development of mHealth solutions for preeclampsia management in LMICs. Ultimately, the anticipated findings of this review are expected to contribute significantly to the understanding of mHealth apps’ role in improving preeclampsia management and addressing healthcare disparities, thereby guiding future strategies to enhance maternal and neonatal health outcomes in LMICs.

## Introduction

### Description of the condition

Preeclampsia is a condition that manifests during pregnancy, typically after the 20th week, and is characterized by high blood pressure and often the presence of protein in the urine [1]. It’s a serious health concern that can lead to complications for both the mother and the unborn child [2]. The exact cause of preeclampsia is not fully understood, but it’s believed to involve several factors, including issues with the placenta, genetic components, and the immune system [3]. Symptoms of preeclampsia can vary but often include rapid weight gain due to fluid retention, abdominal pain, severe headaches, changes in vision, and reduced urine output [4]. These symptoms can escalate quickly and may lead to more severe complications if not managed properly. For the fetus, preeclampsia can result in growth restrictions, preterm birth, and other risks associated with early delivery. Diagnosing preeclampsia involves monitoring blood pressure and testing for protein in the urine [5]. Additional tests may include blood tests to assess liver and kidney function and platelet counts, as well as ultrasounds to monitor the baby’s growth and amniotic fluid levels [6]. Management of preeclampsia typically includes medications to control blood pressure, corticosteroids to help mature the baby’s lungs in case of early delivery, and close monitoring of both mother and baby. In severe cases, the only solution may be to deliver the baby, even if it’s preterm, to prevent life-threatening complications. In Low- and Middle-Income Countries (LMICs), the challenges of managing preeclampsia are compounded by limited access to healthcare, lack of resources, and sometimes cultural practices that may delay seeking medical care [7]. This is where mobile health (mHealth) applications come into play. mHealth apps can help bridge the gap by providing remote monitoring, educational resources, and improved communication between patients and healthcare providers [8]. These apps have the potential to facilitate early detection of high-risk pregnancies and enable timely interventions, which can be crucial in managing preeclampsia effectively [9]. However, the effectiveness of these apps can be influenced by various factors, including the availability of technology, user literacy, and integration with local healthcare systems [10]. Equity is a significant consideration when implementing mHealth solutions in LMICs. Access to technology is not uniform, and disparities based on socioeconomic status, education, and geographic location can affect the utilization of these apps [11]. Ensuring that mHealth apps are accessible and user-friendly for the diverse populations in LMICs is essential for their success in managing [12]. mHealth apps offer a promising solution to improve the management of preeclampsia in LMICs, but they must be designed and implemented with an understanding of the local context and challenges [13]. With the right approach, mHealth apps can contribute significantly to improving maternal and fetal outcomes in preeclampsia cases in LMICs [14].

### Description of the intervention

Mobile health (mHealth) applications are increasingly recognized as a valuable tool for healthcare delivery, particularly in low- and middle-income countries (LMICs) where traditional healthcare resources may be scarce [15]. In the context of preeclampsia management, mHealth apps offer innovative solutions to improve maternal and fetal outcomes by enhancing patient education, facilitating remote monitoring, and improving communication between patients and healthcare providers [9]. Preeclampsia is a serious pregnancy complication characterized by high blood pressure and signs of damage to other organ systems. It poses significant risks to both the mother and the fetus, including the potential for life-threatening complications such as eclampsia, stroke, and organ failure. In LMICs, the prevalence of preeclampsia is particularly concerning due to limited access to prenatal care and a shortage of healthcare professionals trained to manage the condition. mHealth apps designed for preeclampsia management typically include features such as blood pressure and symptom tracking, medication reminders, educational content about the condition, and platforms for communication with healthcare providers [11]. These apps can alert both patients and healthcare workers to potential signs of preeclampsia, prompting timely interventions that can prevent complications and improve outcomes. The effectiveness of mHealth apps in managing maternal health has been demonstrated in various studies [16]. For example, apps that facilitate the use of low-dose aspirin prophylaxis have been associated with improved outcomes in preeclampsia management [14]. Additionally, apps that support community health workers can enhance the delivery of antenatal care and reduce maternal and neonatal mortality. However, the implementation of mHealth apps in LMICs faces several challenges. Technological infrastructure, user literacy, and integration with existing healthcare systems can influence the effectiveness of these apps [17]. Moreover, equity considerations are paramount, as access to mobile technology is not uniform across LMICs. Disparities based on socioeconomic status, education, and geographic location can affect the utilization of mHealth apps, necessitating efforts to ensure that these technologies are accessible and user-friendly for all segments of the population [18].

### How intervention might work

In LMICs, where access to healthcare is often limited, innovative solutions are urgently needed. mHealth apps offer a promising avenue to improve pre-eclampsia management by leveraging mobile technologies for education, monitoring, and communication [11]. Regular monitoring helps prevent complications and ensures optimal outcomes for both mother and child. mHealth apps provide critical effective communication between pregnant women and healthcare, facilitating through enabling secure messaging, appointment reminders and educational content [16]. Such apps can alert patients about warning signs (e.g. Sudden weight gain and severe headaches) and prompt timely consultations. A study has found that an mHealth app improved patient provider communication, aiding in identifying pre-eclampsia risk and promoting a low dose aspirin intake [14]. In rural Guatemala, midwives use mobile health screening tool to identify at-risk pregnant individuals. The app triggers referrals to healthcare facilities, improving maternal and infant outcomes [19]. However, LMICs face unique barriers, including limited infrastructure, language diversity, and cultural norms. These challenges impact app adoption and utilization. Equity considerations are paramount. Access to mobile technology varies, and disparities exist based on socioeconomic status, education, and geographic location. mHealth apps must be designed with inclusivity in mind.

### The importance of the review

The burden of pre-eclampsia in LMICs is high due to a combination of factors, including limited access to healthcare services, lack of awareness, and insufficient resources for timely diagnosis and management [20, 21]. The World Health Organization (WHO) has identified hypertensive induced pregnancy including pre-eclampsia as a leading cause of maternal and perinatal morbidity and mortality, with most of these deaths occurring in LMICs [22]. This highlights the critical need for effective and equitable interventions to manage pre-eclampsia in these regions. mHealth apps have emerged as a promising solution to address the challenges faced in managing preeclampsia in LMICs. They have the potential to improve the accessibility and quality of care for pregnant women at high risk of preeclampsia, thereby reducing the incidence of adverse outcomes. A rapid review of mHealth apps for pre-eclampsia management is essential to evaluate their effectiveness and equity.

## Objectives

To assess the effectiveness and equity of mHealth apps for pre-eclampsia management in low and middle income countries.

## Materials and methods

The search for this rapid review is planned to be conducted on 15th June, 2024. This search will be conducted within one year prior to the expected submission date of the review.

### Criteria for considering studies in this review

#### Types of studies

The review will consider randomized controlled trials (RCTs), quasi-experimental studies, observational studies, and qualitative studies that provide insights into the effectiveness and user experience of mHealth apps for pre-eclampsia. Systematic reviews and meta-analyses that have previously evaluated mHealth interventions for preeclampsia will also be included to synthesize existing evidence. We will limit the inclusion of studies to those published in English at the study selection phase. Based on existing evidence, excluding non-English publications is unlikely to affect the conclusions of clinical intervention reviews [23]. However, if relevant studies in other languages are identified during screening, their inclusion will be considered, and any language-related exclusions will be clearly justified and reported.

#### Types of participants

Participants will include pregnant women diagnosed with or at risk of preeclampsia, healthcare providers involved in the management of preeclampsia, and stakeholders in the development and implementation of mHealth apps for preeclampsia.

#### Types of interventions

*Experimental interventions*. Any mHealth intervention used for the intervention management, monitoring, education, or support of women with or at risk of pre-eclampsia.

*Comparators*. SMS reminders and paper based records.

#### Types of outcome measures

Primary outcomes.

- The Incidence of pre-eclampsia
- Effectiveness of mHealth apps in maintaining or acheiving target blood pressure levels in pregnant women at risk or diagnosed with pre-eclampsia
- Maternal and neonatal morbidity and mortality rates
- User engagement with the apps which can be measured through app usage statistics and adherence to prescribed intervention

Secondary outcomes.

- Improve mother’s quality of life
- Enhanced healthcare utilization interms hospital admissions, antenatal visits, and emergence interventions
- Cost-effectiveness of mHealth interventions, including analysis of healthcare costs and resource utilization
- Increase user satisfaction and acceptance to use mHealth app for preeclampsia management
- Improvements in the user’s knowledge about preeclampsia

### Search methods for identification of studies

The search strategy will be designed to capture a wide range of studies on mHealth applications for preeclampsia management, with a particular focus on those conducted in low- and middle-income countries (LMICs). The search will be as inclusive as possible to ensure that all relevant evidence, published and unpublished, is identified and considered.

### Electronic database searches

The search will begin with electronic databases, which are the primary source of published scientific literature. The databases to be searched will include PubMed, Cochrane Library, Embase, and Scopus. These databases are selected for their extensive coverage of medical and health-related research.

The search strategy will be developed using a combination of keywords and controlled vocabulary terms, such as Medical Subject Headings (MeSH) in PubMed and Emtree in Embase. The search terms will be related to “preeclampsia,” “mHealth,” “mobile health,” “LMICs,” and “management.” Boolean operators (AND, OR, NOT) will be used to combine search terms and refine the search results. Filters will be applied to limit the search to studies published within a specified date range, typically the last ten years, to ensure the relevance and timeliness of the evidence.

**Year Restriction**: The search will be limited to studies published within the last **10 years** to ensure the relevance and timeliness of the evidence.

**Language Restriction**: Initially, no language restrictions will be applied during the search process to capture a broad range of studies. However, during the study selection phase, the review will be limited to **English-language** publications, unless significant studies in other languages are identified as relevant.

### Searching from other sources

- In addition to published literature, the search will extend to grey literature to reduce publication bias and capture data that may not be available in the traditional published sources. Grey literature includes conference abstracts, thesis and dissertation databases, clinical trial registries, and reports from governmental and non-governmental organizations. Specific resources such as the Grey Literature Report and OpenGrey will be utilized. The same search terms used in the electronic database searches will be applied to these sources.
- Handsearching will be conducted for key journals in the field of obstetrics, gynecology, and digital health.
- Consultation with experts in the field of preeclampsia and mHealth will be sought. These experts may provide insights into additional studies, ongoing research, or unpublished data that could be relevant to the review. They may also suggest specific journals, conferences, or databases to search.
- Citation searching, also known as backward and forward searching, will be conducted on key articles identified through the electronic and grey literature searches. This involves reviewing the reference lists of these articles (backward searching) and using citation indices to find subsequent articles that have cited them (forward searching). This method helps to identify additional studies that are related to the topic but may not have been captured through keyword searches.

The search process will be thoroughly documented, including the search terms used, databases searched, the number of records retrieved, and the date of the last search. This documentation will ensure transparency and reproducibility of the search strategy. Given the rapid pace of research in the field of mHealth, the search will be updated periodically throughout the review process to include the most recent studies.

### Data collection and analysis

#### Study selection

Studies will be selected based on predefined inclusion criteria, focusing on randomized controlled trials, observational studies, and qualitative research that evaluate mHealth interventions for preeclampsia management in LMICs. Two independent reviewers will screen the studies to minimize bias and ensure the inclusion of relevant research.

#### Data extraction and management

Data extraction will be organized into five categories

1. **Methods:** The review team will develop a detailed data extraction form that will be used to capture all pertinent information from the included studies. This form will be designed to record the study design (RCT, Non RCT), setting (LMICs), specific methodologies employed (duration, sequence generation, blinding) sample size and analyse in each study. The team will pilot the data extraction form on a small subset of included studies to refine the data collection process and ensure consistency across reviewers.
2. **Population:** Data regarding the population will include demographic details such as age, gender, socioeconomic status, and geographical location. The extraction form will also capture information on the health status of the population, specifically relating to preeclampsia, such as risk factors, severity of the condition, and any comorbidities. This will allow for a thorough understanding of the populations being studied and the generalizability of the findings.
3. **Intervention:** For the intervention category, the data extraction form will include fields to document the type of mHealth app used, its features, the platform it operates on, and the mode of delivery of the intervention. The form will also record the duration of the intervention, the frequency and intensity of use, and adherence to the intervention by participants.
4. **Comparison Groups:** In studies where comparison groups are present, the data extraction form will collect information on the type of control or comparator used. This may include standard care or alternative interventions. The form will also note any differences in the delivery or intensity of the intervention between the groups.
5. **Other:** source of funds, ethical approval

We will filter the abstracts and titles using Covidence or Rayyan. We shall maintain an electronic file outlining the rationale behind the exclusion of potentially relevant research. The data from a few chosen studies will then be extracted using a data extraction form. Data will be extracted from the included papers by two review writers, and any discrepancies will be discussed with the third review author.

### Assessment of risk of bias in included studies

The assessment of risk of bias in included studies will be a critical component to ensure the validity and reliability of the findings. This assessment will be conducted using a structured approach that will encompass several key domains known to influence the risk of bias in the research. The review team will employ a comprehensive tool for assessing the risk of bias in each included study. This tool will be based on the Cochrane Collaboration’s recommendations and will involve a judgement and a support for the judgement for each entry in a ‘Risk of bias’ table [24].

For each study, the reviewers will assess the risk of bias as ‘low risk’, ‘high risk’, or ‘unclear risk’. The ‘unclear risk’ category will indicate either lack of information or uncertainty over the potential for bias.

The domains that will be addressed in the risk of bias assessment will include, but not be limited to, random sequence generation, allocation concealment, blinding of participants and personnel, blinding of outcome assessment, incomplete outcome data, selective reporting, and other sources of bias. The reviewers will check for incomplete outcome data and whether it was addressed correctly in the studies. They will evaluate if the missing data could significantly bias the results.

Selective reporting of outcomes will be scrutinized. The team will investigate whether the study protocols are available and if the reported findings align with the pre-specified outcomes.

Other sources of bias will be considered, including the funding source, conflicts of interest, and any other factors that could introduce systematic errors into the study results. The findings from the risk of bias assessment will be incorporated into the review’s results and will be used to inform the conclusions drawn from the evidence. The overall risk of bias for each outcome will be summarized to provide a clear picture of the validity of the findings. The assessment of risk of bias will be reported transparently, following the Preferred Reporting Items for Systematic Reviews and Meta-Analyses (PRISMA) guidelines [25], to facilitate the replication and verification of the review process.

### Measures of treatment effect

The review will incorporate a variety of effect measures to evaluate the outcomes of mHealth interventions. These measures will be chosen based on the type of data available from the included studies. For dichotomous outcomes, such as the incidence of severe preeclampsia complications, the review will calculate risk ratios (RR), odds ratios (OR), and risk differences (RD). For continuous outcomes, such as changes in maternal blood pressure, the review will use mean differences (MD) when outcomes are measured in the same way across studies.

The number needed to treat (NNT) will also be considered for inclusion as a measure of treatment effect. This statistic will convey the number of patients who need to be treated with the mHealth intervention, as opposed to the control, to prevent one additional adverse outcome. Time-to-event data, which may include the time until a significant drop in blood pressure is achieved, will be analyzed using hazard ratios (HR). This measure will account for the fact that not all participants may experience the event during the study period.

The review will ensure that ratio measures, such as RR and OR, will be analyzed on a logarithmic scale to stabilize variances and normalize the distribution. This approach will facilitate more accurate statistical analysis and interpretation of the results. The review will also consider the precision of the effect estimates by calculating confidence intervals (CI) for each measure. The CI will provide a range of values within which the true effect size is expected to lie with a certain level of confidence. In cases where meta-analysis is appropriate and feasible, the review will combine the results of individual studies to produce an overall estimate of the treatment effect. This will be done using either a fixed-effect or random-effects model, depending on the level of heterogeneity observed among the study results.

For qualitative studies, thematic analysis will be performed to synthesize findings, focusing on user experiences, barriers, and facilitators associated with mHealth interventions. The qualitative data will be integrated with the quantitative results to provide a comprehensive understanding of the interventions’ effectiveness and context.

### Unit of analysis issues

The authors will identify and address unit of analysis errors, which occur when individual patient data are analyzed as if there was no clustering within randomized groups. They will ensure that the number of observations in the analysis matches the number of units that were randomized.

### Dealing with missing data

Investigators will handle missing data by employing various methods such as multiple imputation or sensitivity analysis to assess the impact of missing data on the study’s conclusions. They will make explicit the assumptions of any methods used to cope with missing data.

### Assessment of heterogeneity

Study designers will assess heterogeneity, which is the variability among study results, by examining clinical diversity, methodological diversity, and statistical heterogeneity. They will use measures like the I^2^ statistic to quantify the degree of heterogeneity observed in the meta-analysis.

### Assessment of reporting biases

Future reviews will include an assessment of reporting biases, such as publication bias and selective reporting, using tools that evaluate the risk of such biases in studies and syntheses of studies. They will ensure transparency in how these biases are handled within the review.

### Subgroup analysis and investigation of heterogeneity

Authors will conduct subgroup analyses to investigate heterogeneous results or to answer specific questions about particular patient groups, types of intervention, or types of study. They will explore potential sources of heterogeneity and adjust their analyses accordingly.

### Sensitivity analysis

Sensitivity analyses will be performed to assess the robustness of the primary analysis conclusions under different assumptions or scenarios. This will include repeating the primary analysis with alternative decisions or ranges of values for decisions that were arbitrary or unclear.

### Overall quality of body of evidence

The overall quality of the body of evidence will be graded based on several domains relating to the quality of published studies and the consistency and other characteristics of findings from those studies. The strength of evidence will be categorized as high, moderate, low, or insufficient.

### Summary of findings table

The summary of findings table will present the primary findings of a review in a structured tabular format, particularly information related to the quality of evidence, the magnitude of the effects of the studied interventions, and the aggregate of available data on the main outcomes.

Each of these components will be meticulously planned and executed to ensure the integrity and reliability of the systematic review. The researchers will document their methodologies and findings with clarity and precision, contributing to the body of knowledge in the medical field.

## Discussion

The purpose of this review is to assess the effectiveness and equity of mHealth apps for the management of pre-eclampsia in low and middle income countries (LMICs). The use of mHealth apps is a promising solution in LMICs where healthcare infrastructure is often underdeveloped [26–28]. These apps offer critical tools for monitoring, education, and communication, which can aid in the early detection and management of preeclampsia. Effective management of this condition could reduce both maternal and neonatal morbidity and mortality, particularly in resource-constrained settings [29].

This review has the potential to significantly impact both clinical practice and policy by providing a comprehensive analysis of the effectiveness and equity of mHealth applications for managing preeclampsia in low- and middle-income countries (LMICs). The results could offer evidence-based recommendations on how mHealth solutions can enhance pre-eclampsia care in areas where healthcare access is limited. By integrating mHealth into preeclampsia management, the review highlights the opportunity to empower women with tools to monitor their health, improve early detection, and potentially reduce complications, ultimately lowering maternal and neonatal morbidity and mortality rates. Policymakers and healthcare practitioners could use these insights to promote scalable digital interventions that make healthcare more accessible and personalized.

However, key potential challenges that may arise include the digital divide, as unequal access to smartphones, mobile networks, and reliable internet connections that may limit the reach of mHealth applications, especially in remote or economically disadvantaged areas. This could exacerbate health disparities instead of reducing them. Additionally, cultural and societal factors may impact the adoption of digital solutions, with certain communities being resistant to using mHealth technology or having concerns about its efficacy. Another challenge lies in the integration of mHealth tools into existing healthcare systems, as healthcare providers need training and support to use these applications effectively. Moreover, there may be challenges in ensuring that these technologies are used in a manner consistent with local health policies and aligned with existing practices, requiring strong coordination between digital health developers and local health authorities.

We anticipate that the results of the review will contribute to the growing body of literature advocating for the integration of technology in healthcare delivery, particularly in resource-limited settings. By highlighting successful mHealth initiatives, this review can inspire further investments and innovations in mobile health technologies, fostering collaborations between governments, NGOs, and private sectors to enhance maternal health services.

## Supporting information

S1 File Prisma_P_Checklist. (DOCX)
